# Semiparametric Multinomial Ordinal Model to Analyze Spatial Patterns of Child Birth Weight in Nigeria

**DOI:** 10.3390/ijerph13111145

**Published:** 2016-11-17

**Authors:** Rasheed A. Adeyemi, Temesgen Zewotir, Shaun Ramroop

**Affiliations:** 1 School of Mathematics, Statistics and Computer Science, University of KwaZulu-Natal, Pietermaritzburg Campus, Private Bag X01, Scottsvile 3209, South Africa; ramroops@ukzn.ac.za; 2 School of Mathematics, Statistics and Computer Science, University of KwaZulu-Natal, Westvile Campus, Durban 4000, South Africa; zewotir@ukzn.ac.za

**Keywords:** Nigeria, child birth size, cumulative multinomial model, penalized spline, spatial maps

## Abstract

*Background*: Birth weight is an important health parameter for obstetricians and gynaecologists. It is a good health indicator of a child-bearing mother and a strong predictor of infant morbidity and mortality. *Methods*: This paper utilizes data on 28,647 children born between 2003–2008 obtained from the 2008 Nigeria Demographic and Health Survey (NDHS). For a simple epidemiological convenience, the occurrence of a newborn weight can intuitively be considered to be categorical in nature and the thresholds can be put on a continuous scale. In survey reporting, the mothers frequently estimate their infant’s birth weight and make a classification in ordinal category (low, normal, large) instead of actual birth weight. The study fits a multinomial regression model to analyze the relationships between the polytomous response and different kind of covariates in a unified manner. We estimate the fixed effects of bio-social covariates parametrically and the non-linear effect modeled using P-spline. The spatial component was modeled using conditional autoregressive error. A penalized maximum likelihood estimation was performed to estimate the model parameters. *Results*: We found risk factors that are positively associated with low birth weight, which include multiple birth, short birth interval, death of sibling, childhood diarrhea, fever, mother’s smoking, firewood/dung cooking and poor household. Results further showed that iron syrup supplementation, antenatal attendance, mother literacy and household wealth had significant association with low probability of low birth weight. The finding also showed spatial patterns, which are not captured by the underlying determinants, and we produced probability predictive maps of the spatial residual effects. *Conclusions*: In addition to the statistical relevance of our method, the generated spatial maps identify highly endemic areas of low birth weight that can assist government agency to channel scarce health resources. A comprehensive approach which institutes a combination of interventions to improve the overall health care of the women is needed.

## 1. Introduction

Birth weight is the result of fetal growth and a good predictor of infant morbidity and mortality. Birth weight is a strong indicator not only of the mother’s health but also on a newborn’s chances of survival, growth, long term health and psychological development [1]. In the last 3 decades, there has been increasing evidence that birth weight and pregnancy complications are independently associated with the increased risk of mortality and early morbidity in babies, as well as poor maternal health outcomes [2,3,4]. In 2015, an estimated 303,000 maternal deaths occurred due to pregnancy complications during childbirth and 5.9 million children are under-five age died, of which 45% of these deaths occurred within the first 28 days of their life [5].

For a simple epidemiological interpretation, low birth weight (LBW) is defined as a birth weight <2500 g and it has remained a significant health problem in many parts of the world due to immediate and long-term consequences [6]. Reports have showed that about one-half of all LBW infants outcomes in industrialized countries are born preterm (<37 weeks gestation), most LWB infants in developing countries are affected by intrauterine growth restriction that may begin early in pregnancy [7,8].

A number of studies has investigated the impacts of socio-economic characteristics of mothers on their newborns birth weight. Scanty research work has been done that jointly underscores the influence of the geographical variations in mother’s location and the underlying determinants of birth weight. In this study, we propose a structured additive regression model to examine the influence of different covariates on a categorical response variable. The motivation of this study is the ability of our approach to analyze a multivariate natural ordered response and simultaneously handle covariates of different types in a unified framework. We also explore a small area estimation of the spatial residual effects in child birth weight that are not captured by the underlying factors, which would have been neglected in the classical regression approach.

The paper is structured into five sections. In Section 2, we discuss the sampling procedure and statistical methods. Section 3 presents the results of the data analysis, while Section 4 discusses the results. In Section 5, the concluding remarks are given.

## 2. Methods

### 2.1. Sampling Survey and Data

One of the reliable sources of national household-level data on fertility, family planing and children nutritional status are the Demographic and Health Surveys (DHS). The 2008 Nigeria DHS (NDHS) was designed to provide, among other things, information on maternal health, prenatal and postnatal care, altitudes and micronutrient supplementation during pregnancy. The survey covers a national representation of women of reproductive age (15–49 years) and NDHS includes data on birth weights measured on their children aged 1–59 months. Other variables relate to information on levels and trends in fertility; sexual partners; fertility preferences; awareness and use of family planning methods; infants and young children feeding practices; nutrition status of mothers and childhood mortality. However, maternal health outcomes such as child birth weight plays a vital role in the understanding the epidemiology of public health problems, children’s growth restriction, infant mortality trend and childhood survival. The detailed information about the sampling techniques of the survey have been published in the final report of National Population Commission and ICF Macro [9].

**Figure 1:**
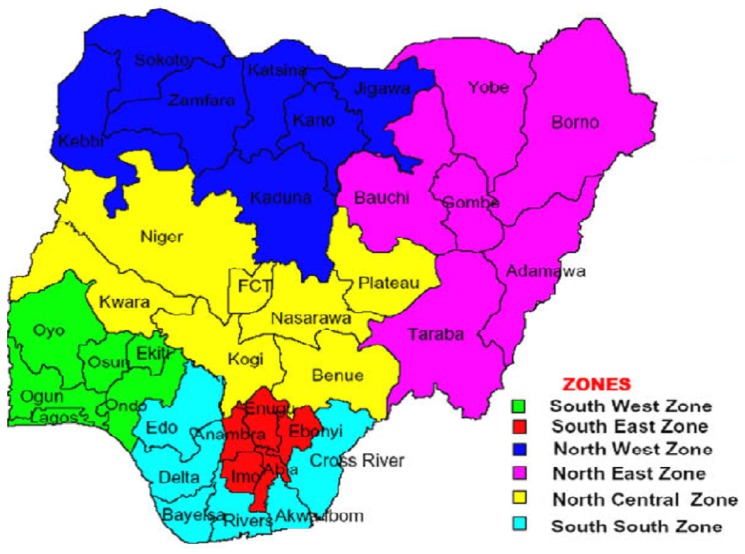
Map of Nigeria showing 36 states (districts) and Federal Capital Territory (FCT) by geopolitical zones.

Nigeria is made up of six geopolitical zones and subdivided into 36 states and the Federal Capital Territory (FCT), as the second administrative levels. Figure 1 displays the map of Nigeria showing states grouped by geopolitical zones. The states and FCT are further subdivided into local government areas, as the third level of administration. NDHS-2008 utilizes a two-stage probability sampling. At the first stage, 888 clusters consisting of 286 in urban and 602 in rural areas were selected from the sampling frame as used during the 2006 National Population Census (NPC). The primary sampling unit (PSU), known as a cluster in the 2008 NDHS, is defined on the basis of an Enumeration Areas (EA) according to the 2006 NPC. The sample frame of households in each selected cluster was obtained and the households were randomly drawn for interviewing. At the second stage, a sample representative consisting of 36,800 households is randomly drawn for an interview with a minimum target of 950 interviews per state and in at least 41 clusters. In each state, the number of households selected for interview is proportionally distributed among urban and rural areas. Interviews were conducted for 33,385 women age 15–49 and 15,486 men ages 15–59, resulting in a total response rate of 97% interviewed.

### 2.2. Model Formulations

This study utilizes data obtained from the 2008 Nigeria Demography Health Survey (NDHS). The data set on child birth was generated with the aim of assessing the influence of some covariates on the response (child birth size) as reported in the health surveys. NDHS data set contains several other variables, only those that are related to child birth weight and those similar to the ones considered in the descriptive analysis were selected. The children involved in the survey had ages ranging between 1–59 months and the respondents (mothers) are in reproductive age range 15–49.

The response variable of interest (child birth weight) is classified as

$$\mathrm{Model}A\;\;\;\;y_{i1}=\left\{\begin{array}{cc} 1 & \mathrm{if}\;\mathrm{the}\;\mathrm{child}\;\mathrm{birth}\;\mathrm{weight}\;\mathrm{is}\;\leq 2500\;g\\ 0 & \mathrm{otherwise} \end{array}\right.$$

A multi-categorical representation of the response variable is coded as

$$\mathrm{Model}B\;\;\;y_{i2}=\left\{\begin{array}{cc} 1: & \mathrm{smal}\;\mathrm{size}\;,\mathrm{if}\;\mathrm{the}\;\mathrm{cbw}\;\mathrm{is}\;<2500\;g\\ 2: & \mathrm{average},\;\mathrm{ifthecbwis}\;\mathrm{the}\;\mathrm{cbw}\;\mathrm{is}\;\geq 2.5\;\&<3.2\mathrm{kg}\\ 3: & \mathrm{large},\;\mathrm{if}\;\mathrm{the}\;\mathrm{cbw}\;\mathrm{is}\;\geq 3.2\&<4.0\mathrm{kg}\\ 4: & \mathrm{very}\;\mathrm{large},\;\mathrm{if}\;\mathrm{the}\;\mathrm{cbw}\;\mathrm{is}\;\geq 4.0\mathrm{kg} \end{array}\right.$$

where $y_{i1}$ is binary response outcome and $y_{i2}$ is an ordered categorical response outcome. The present study intends to apply a flexible regression model to quantify the effects of fixed and non-linear factors as well as geographical variations of the child birth size as response variables $y_{i1}$ and $y_{i2}$ as defined above.

### 2.3. Model Specification

We propose a structured additive regression model to examine the impact of different types of covariates on child birth weight. The parameters in the model are estimated in a unified regression model. We employ *BayesX 2.0.1* version software for modeling structured additive regression developed by Brezger et al. [10] via penalized maximum likelihood (PMLE) methods. With a varying combination of covariates, we formulate three different model specifications:

**M** **1** η= Spatial + random effects (No Covariates)
**M** **2** η= Linear effects ONLY (Linear model)
**M** **3** η= Non-linear and Linear effects + Spatial effects (Geoadditive model)

### 2.4. Multinomial Logit Models

In recent decades, there has been growing interest in the application of an ordinal logistic regression model and its transformation into a latent variable model [11,12,13]. Such regression models based on multi-categorical outcomes are sometimes called cumulative regression models, and its distrubtional form had been previously investigated in the literature [14,15]. The models can be motivated from latent variables such that the response variable *Y*, say, birth weight, can be written as a categorical ordered response of a continuous latent (utility) variable

(1) Z=η+ε

where *η* is a predictor depending on covariates and parameters and *ε* is the error term. The two variables *Y* and *Z* are linked by Y=j if and only if

(2) $$\theta_{j-1}<Z\leq \theta_{j},\;\;\;j=1,2,3,4$$

with thresholds $-\infty <\theta_{0}<\theta_{1}<\ldots <\theta_{k}=\infty$. In a multinomial logit model setting, the error variables *ε* in (1) are independent across the categories and assumed to be standard extreme value distributed with function *F*. Hence, it follows that *Y* obeys a cumulative model. The predictor is then defined as

(3) $$Pr\left(y_{i}\leq j|\eta \right)=F\left(\theta_{j}-\eta \right)$$

For identifiability, the linear combination does not contain an intercept term $\gamma_{0}$, otherwise one of the thresholds must be set to zero. The probability of selecting $j^{th}$ birth category as against the reference category, say, very large birth size, is computed by the multinomial logit model given as

(4) $$Pr\left(y_{i}=j|y_{i}\geq j,\eta_{i}\right)=\frac{\exp(\theta_{j}-\eta_{i})}{1+\exp(\theta_{j}-\eta_{i})}=F\left(\theta_{j}-\eta_{i}\right)$$

Consider a set of regression observations $(y_{i},x_{i},s_{i},v_{i}),i=1,2,\ldots ,n$, where $y_{i}$ is either binary or categorical response variable, a vector $x_{i}$ is the of metrical covariate effects of the mother’s age at birth and body mass index, the spatial covariate $s_{i}\in \left[1,\ldots ,37\right]$ of the district (state) where mother *i* lives in Nigeria and a further vector $v=(v_{i1},\ldots ,v_{iq})$ of categorical covariates. For a model B as specified above, we propose a semiparametric predictor as suggested by [13] defined as

(5) $$\eta_{i}=\theta_{i}^{j}-\left(f\left(x_{i}\right)+f_{spat}\left(s_{i}\right)+v_{i}^{\prime }\gamma \right)$$

where, $f(x_{i})$ and $f_{spat}\left(s_{i}\right)$ represent the estimates of the unknown non-linear smoothing effects of the metrical covariates $x_{i}$ and the spatial effect respectively, while $v_{i}$ is a vector of the fixed (categorical) effect. The spatial component, $f_{spat}\left(s_{i}\right)$ captures the random effects of area $s_{i},s\in \left\{1,\ldots ,37\right\}$, where the woman *i* resides. The spatial component, $f_{spat}\left(s_{i}\right)$ is further split into two components: $f_{str}\left(s_{i}\right)$ and $f_{unstr}\left(s_{i}\right)$ as spatially structured (correlated) and unstructured (uncorrelated) random effects, respectively.

### 2.5. Estimation Procedure

Within appropriate re-parameterization, the semiparametric multinomial logit Model (5) can be rewritten as a mixed effect formulation as suggested in [16,17,18] of the predictor and estimation are done via a penalized likelihood approach. The smooth effects functions and model parameters are then estimated simultaneously for the regression coefficients. Inference is therefore achieved with empirical Bayes approach based on generalized linear mixed model (GLMM) methodology by partitioning the coefficients into a penalized and unpenalized part yielding a variance component model [18,19]. Based on the GLMM formulation approach, regression and variance components can be estimated using an iterative weighted least squares (IWLS) and restricted maximum likelihood (REML) developed for GLMM [18,20].

## 3. Statistical Analysis

### 3.1. Data Processing

The data was extracted from the 2008 NDHS and checked for completeness and missing values. It was found that the proportion of missing values and non-response was negligible. The data was cleaned and re-coded in R for further exploratory analysis. Summary statistics such as mean and standard deviation were computed for continuous covariates considered in the models. The percentages of categorical cases were computed using R and multivariate (geoadditive) regression analysis was performed using BayesX version 2.1 via a penalized maximum likelihood estimation as suggested by Fahrmer et al. [18].

### 3.2. Preliminary Analysis

The selection of explanatory variables was done on the basis of previous empirical studies [21,22] among others. In the original 2008 NDHS, the mother’s estimation of the size of her baby at birth was asked for children born in five years before the survey. The birth size was given in five scale categories: very small, small, average, larger than average, and very large. In this report, very small and small are combined into one category to represent the detrimental birth outcome. The other three categories are considered non-detrimental. The response variable is constructed by categorizing the child birth size into four-ways by (low, average (normal), large, very large) and the percentage distribution presented in the Table 1. This type of categorization is consistent with previous report [23,24]. The percentages computed for the four-way birth category were low size, 4239 (14.82%), larger than average 7852 (27.4%), very much larger than average, 5160 (18%) and normal size 10,732 (37.5%). The total number of births in 2008 NDHS classified by sizes was 27,983 (97%) children out of total live births of 28,647 births.

The categorical covariates are grouped into two broad categories according to birth sizes. The bivariate analysis that used $\chi^{2}$-statistic test is presented in Table 2. The variables are derived from child and mother characteristics, as well as environmental factors, e.g., place of residence and childhood diseases (diarrhea, fever, cough) and nutrition status of the child, for further analysis. The analysis showed that all the covariate groups differ significantly in their proportional distribution in the two-way classification of birth size.

A four-way classification of the birth weight distribution was presented in Table 3. It can be observed that the proportion of low weight deliveries exhibits an inverse relationship with the birth order 1–4, except order 5 (5 + order). Further, the low birth deliveries also decline linearly with rise in maternal educational levels. Further, the low birth weight also shows an inverse linear relationship with increase in household wealth index. In other words, the household wealth index can be expressed as an inverse association with low birth weight. It is also noteworthy that there are differential variations in patterns across the six geopolitical zones. The highest incidence of low birth weights were recorded in the North East and North West regions of the country.

Table 3 presents the summary of the descriptive analysis of social and demographic characteristics of the households interviewed in the survey, which is categorized into four-way child birth size. The percentage distribution in Table 3 is similar to Table 2 and includes other categories. Analysis in Table 3 shows that a higher proportion of the women did not attend antenatal classes. Also, the proportion of women, who did not attend antenatal checks were higher than those who attended, which cuts across the four birth strata. About half of these women (48%) received iron syrup supplementation during pregnancy. Of these women, 77 percent of the children they delivered (within the last five year) did not receive postnatal vitamin A supplementation. The analysis further showed that a substantial percentage of the children who were born with low birth weight also suffer from childhood under-nutrition (such as stunting and wasting). It can observed that the incidence of malnutrition declines linearly as the birth size increases (i.e., horizontally). The descriptive statistics in Table 4 also includes mean and standard deviations of some metrical covariates, considered in the multivariate analysis.

### 3.3. Results of Multivariate Analysis

In the model B.1, we fit a spatial semi-parametric model for the response category (birth size) distribution of spatial random effects without a covariate. For the model B.3, we fit a spatial semi-parametric model consisting of spatial effects, linear and non-linear covariates. The categorical covariates (environment factor, mother and child characteristics) as fixed effects and geographical location of the woman were simultaneously estimated on the response variable (birth weight). The outputs of the analysis are presented in the form of Tables, non-linear graphics and spatial maps.

#### 3.3.1. Model Fit

In this section, we present the results of model selection criteria values of six fitted models. The model selection criteria and their values for the models are presented in Table 5. Our model selection strategy is as follows: to convey the potential extreme distributions, we consider a base model (Model 1) without covariate, which includes only the spatial effect components and a full Model (Model 3). Model 3 includes the spatial effects and other covariates. The continuous variables are modeled using P-smooth splines, the categorical covariates using dummy variables (one represent a factor level , −1 as reference, 0 other level) and the geo-spatial components are modeled by a Markov random field, whereas Model 2 includes only fixed (categorical) effects.

On the binary logit model, we realized the AIC = 22,520.7 for the model without covariates (Model A.1), linear (AIC= 5042.1) and AIC = 6163.13 for the full Model (A.3). On the cumulative multinomial logit model, we obtained AIC = 72,630.0 for the model without the covariates (B.1), and AIC = 23,667.2 for the geoadditive model B.3. In both cases, we conclude that the model with the covariates effects is a better than the one without any covariate. In addition to the Akaike Information Criterion (AIC), we provided the values of Bayesian Information Criterion (BIC), which emphasizes on the model complexity than the simplicity. AIC relies on model likelihood from the data and the number of parameters estimated in the model. However, our Models 1 and 3 include spatial structure of the geographical location of the woman (respondent), and such models are more complex than the linear Model 2. In other words, the Generalized Cross Validation (GCV) criterion emphasizes on the model optimality rather than simplicity and complexity explores by the former two criteria. The researcher is left with varying options in selecting the preferred model.

The results of the analysis are presented in form of Tables of fixed (categorical) effects, non-linear plots of continuous covariates and the structured residual plots of spatial components for the binary logit and cumulative multinomial model.

#### 3.3.2. Fixed Effects

Table 6 presents the posterior estimates of fixed effects of the socio-demographics variables considered in the binary logit model. A careful interpretation of the coefficient signs of these estimates and the 95% confidence interval is observed. The 95% credible intervals include zero. This means the effect is not statistically significant at the 5% probability level. From the binary logit analysis, the hypothetical results showed that the predictor quantifies the impact of the variable on low birth size. A negative coefficient indicates that the effect of the variable (factor) would improve the outcome of the low birth weight (LBW) compared to the reference factor, while a positive coefficient of a variate worsens the low birth rate. The signs of the coefficient estimates for the models A.2 (linear) and A.3 (geoadditive) look similar. It can be seen that A.2 is a submodel of A.3. The interpretation would be based on model A.3.

The results showed that there was evidence of significant association with higher probability of low birth weight in the North East compared with the North Central region. This indicates that women in the North East had higher odds of low birth weight incidence or higher risk of low birth size than the North Central zone. All other geopolitical zones indicate lesser likelihood of low birth size but these were not statistically significant. Marginally, the Southern Zones had lower prevalence of low birth sizes when compared with the Northern parts of Nigeria.

The results from Table 6 (column Models A.2 and A.3) further showed that a male child had a higher probability of being born with low birth weight and a child born from a multiple (twin) birth also had higher risk of low birth weight than a single birth. This implies that a male child had a higher risk of being born with a low birth weight compared to a female counterpart, while a male child raised the likelihood of LBW by 16% compared with a female child and a child from a multiple birth would increase the risk of LBW by 45% compared with a single birth. A short birth interval (2 or more children within 3 years) also showed a significantly higher probability of low birth weight compared with a well spaced birth interval, indicating that a short birth interval increases the odds of LBW by 17%, OR = 1.17, 95% CI (1.07, 1.29) compared with a single child birth.

In addition, environmental factors include poor household, death of sibling 2 and 4, firewood/ dung method of cooking and mother smoking, childhood diseases (diarrhea and fever 2 weeks before the survey) were found to contribute to the higher risk of LBW, although they were not statistically significant at 5% probability level. A child born of very low birth weight had a high risk of being susceptible (low immune)to child diseases in their early years of life. There was significant association between childhood under-nutrition (wasting) and low birth weight. The result showed that children born of low birth weight would suffer of growth restriction in their early life (infancy).

There was evidence that micro-nutrient intervention such as iron syrup supplementation during pregnancy would boost low birth weight. Also, postnatal vitamin A to children would boost the growth of child born of low birth weight, although vitamin A supplementation was not significant. Iron supplementation to pregnant mother was significant and reduced odd of LBW by 21%, i.e., OR = 0.79, 95% CI (0.73, 0.86), compared to those mothers, who did not take iron syrups. Mother, who attended antenatal also had lesser likelihood of giving birth to baby with low birth weight.

The result further showed a strong impact of mother characteristics for improving her birth outcome. Maternal literacy (ability to read or write), urban residence mother, mother belong to richer household would likely would reduce their chances of low birth weight. A literate mother would have a lesser probability (reduce odd) of giving birth to baby with LBW by 11%, i.e., OR = 0.89, 95% CI (0.82, 0.96) compared to an illiterate mother.

Further, similar influence of covariates variables were observed from the multinomial model as presented in Table 7. The model B.2 (linear model) can be considered as a sub-model of model B.3. The parameter signs of the variables in the models are similar. From the results on model B.3, the estimates include the threshold parameters $\theta_{1},\theta_{2}$ and $\theta_{3}$ and other categorical variables estimated. We interpret threshold parameters as a lower (higher) value corresponds to less (higher) likelihood of the birth weights category shifting on the latent scale. For instance, a negative sign of $\theta_{1}$ and $\theta_{2}$ signifies a shift on the latent scale to the left side, yielding a lower probability for category of low birth and average size categories respectively. This can be interpreted in terms of the relative odds of shifting from higher category (very large) to low birth outcome. For instance the relative odds of shifting from very large to low birth size per unit increase in the predictors is given by $\theta_{1}$, shifting from low birth to normal size for $\theta_{2}$, shifting from normal to large birth size for $\theta_{3}$. This can be stated as the relative odds of very large of a low birth size, (exp(1.334)), i.e., about 4 times as high as obtaining a very large birth size, and 2.028 times for get low size to normal (average) birth size. Similarly, a positive sign of $\theta_{3}$ signifies a shift on the latent scale to the right side, yielding a higher probability for the larger category. Putting in mid that that the reference category was very large size.

We further explore the estimates on the model B.3 in Table 7, iron syrup supplementation to mothers during pregnancy would significantly increase the relative odds of larger birth size than those mothers with no supplements. Mother literacy was found to be significant with relative higher likelihood of large size to low birth size for non-literate mother. There was significant evidence that mother firewood/dung cooking method would significantly reduce the relative odds of the larger birth size to low birth size over gas/kerosene cooking.

#### 3.3.3. Non-Linear Effects

**Figure 2:**
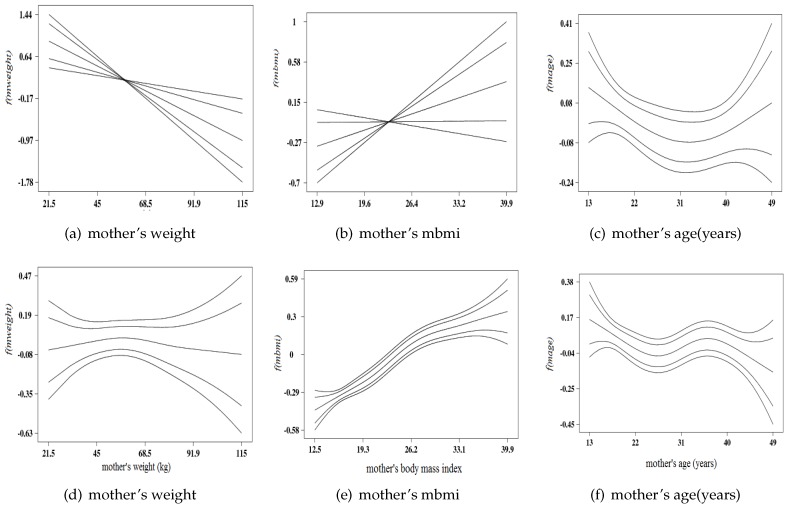
Non-linear effects of (**a**) Mother’s weight; (**b**) mother’s body mass index; and (**c**) mother’s age at birth **Model A** Binary logit model in *upper panel* (**d**) mother’s weight; (**e**) mother’s body mass index; and (**f**) mother’s age at birth for **Model B**, Multinomial model on *lower panel*.

The non-linear effects components of the metrical (continuous) covariates in the binary logit model A.3 (in Table 6) are displayed in the upper panel of Figure 2, while lower panel consists of graphs of model B.3. Each plot in Figure 2 is made up of 5 trend lines with the center line is the smooth estimate of the posterior mean bounded by the two inner lines are 95% credible intervals and outer lines are 80% credible intervals.

Observing from the upper panel, Figure 2a,b depict linear functions with Figure 2a shows a inverse relationship of the smooth effect of the mother’s weight on the child birth weight as estimated from the binary logit model. The graph depicts that an increase in mother’s weight would reduce the chance of low birth weight. We noticed a consistent linear association between the smooth effect the mother weight increases as shown in the Figure 2a. That is the heavier mother reverses the possibility of giving birth to baby with low birth weight.

Further results from the binary logistic model, Figure 2b showed that the effect of mother’s body mass index (mbmi) = weight(kg)/height${}^{2}$(m) on the low birth weight. It can be observed that the graph depicts a discernible relationship between ’mbmi’ and her baby birth weight. A threshold points can be invoked according the World health Organization (WHO). The plot can be factored into 3 parts: below 18.5, (as underweight mother) would increase the odds of low birth size (underweight) and mbmi above 24.5, overweight mother would increase the likelihood of large size babies, their babies are at the risk of obesity.

Figure 2c illustrates the plot of the effects mother’s age on the low birth weight. The plot depicts a U-shape function. The graph can be segmented into 3 parts (thresholds) as the effect of mother’s age below 20 years, (teenage mother) would increase the likelihood of low birth weight (downward trend), mother age between 20–40 years stabilizes the effect on birth size, and mother age above 40 years would improve the probability of baby birth size.

On the lower panel of Figure 2 displayed the non-linear effects of three (3) metrical covariates in multinomial model B.3. Figure 2d show the non-linear effect of mother’s weight showing inverted U-shape. This implies that for birth size and mother’s weight, indicating an inverted U-shape and Figure 2e depicts a S-shape function, known as sigmoid function. This represents the impact of mother’s nutritional status on her child birth weight and Figure 2f the effect of mother’s age at birth on her child birth weight. The resulting trend possess a discernible non-linear relationship. In other word, one can deduce that a teenage mother (<20 years) resulting in a downward trend on her baby’s birth weight, mother’s age between 20–40 years would lead to an upward concave curve and mother above 40 years would result into a downward concave on her baby’s birth weight.

#### 3.3.4. Spatial Effects

**Figure 3:**
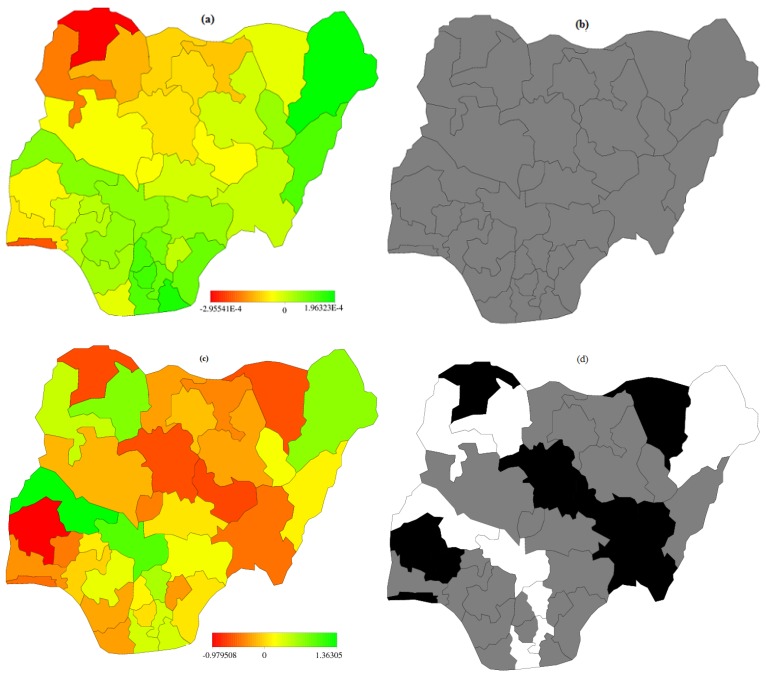
Spatial effects of (**a**) posterior mode and (**b**) 95% credible intervals of model A.1 (**c**) posterior mode and (**d**) 95% credible intervals of Model A.3 for binary logit model.

**Figure 4:**
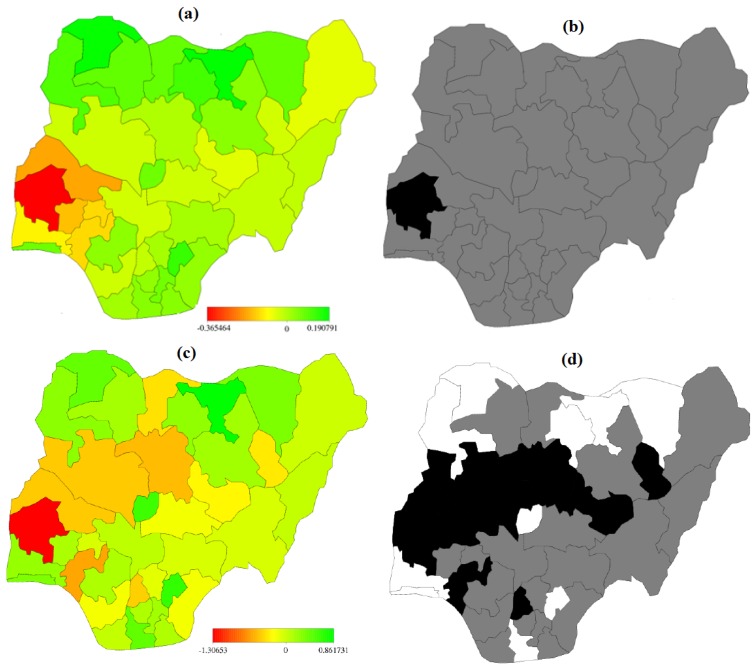
Spatial effects of (**a**) posterior mode and (**b**) 95% credible intervals of model B.1 (**c**) posterior mode and (**d**) 95% credible intervals of Model B.3 for cumulative multinomial model.

Spatial effects for the two geoadditive binary logistic models are presented in Figure 3a–d and two (2) cumulative multinomial Figure 4a–d. Presented are maps of posterior modes *(left panel)* and the maps showing the 95% credible intervals *(right panel)* are used to determining the significance level. Using 95% credible intervals, states with white (black) colours are associated with significantly high (low) estimates corresponding to regions lie in the positive (negative) sides, while the grey colour depicts the estimates are not significant among states.

The 95% credible intervals from Figure 3b, shows that all states are “grey” coloured, this depicts that there were no significant variation. For model A.3, Figure 3d, there were evidence of spatial variations in the birth weight sizes across the states after adjusting for some confounding factors. It can be observed from Figure 3d that the states with black colour are strictly negative, indicating a low probability of low birth sizes in those states. The states include Lagos, Oyo, Sokoto, Kaduna, Plateau, Taraba and Yobe. The ‘white coloured’ regions are strictly positive, these states are associated with high prevalence of low birth size. The states are; Rivers, Abia, Enugu, Kogi, Kwara, Kebbi, Zamfara and Borno. The high incidence of low birth weight in those states might be connected with manual labour and farming activities engaged by the women.

Figure 4 displayed the spatial components for the multinomial Models B.1 and B.3. The left panels (a & c) are the posterior means of residual spatial effects showing evidence of spatial variation. Using Figure 4b,d to determine the significance level, Figure 4b shows that Oyo state with black colour had a relative low probability of shifting from very large to low birth weight. Other states with grey coloured had no significant variation. With Figure 4d, the black coloured regions are associated with relative low probability (strictly negative) of shifting from very large birth size (reference category) to low birth sizes. The states are: Ondo, Oyo, Kwara, Niger, Kaduna, Plateau and Gombe. The white coloured regions are associated with high likelihood of shifting from very large birth size (reference category) to low birth sizes. These records are observed at states: Lagos, Ogun, FCT-Abuja, Rivers, Ebonyi, Kebbi, Sokoto, kano, Jigawa and Yobe. Perhaps, this trend might be attributed to growing population in cities coupled with over stretched facilities. Other regions with grey coloured are not significant.

## 4. Discussion

This study investigates the social and demographic (environmental, maternal and child characteristics) impacts on the child birth weight. Our approach also assess the geographical variations in child birth weight across the states and the woman likelihood of having a child birth weight falling in a specific category. Our fixed effect estimates of regression models look reasonable and controlled for all stable characteristics of the mother including household wealth index and child-specific factors. The descriptive analysis showed that low birth weight delivery decreases with an increase in birth orders of second , third and fourth as compared to first order births, but not to the fifth order (5 or more). The incidence of low birth weight are inversely associated the household wealth index. The bivariate analysis showed that a large proportion of women did not attend antenatal and they did not give postnatal vitamin A supplements to their children.

From the binary logit analysis, the findings revealed that mother literate and prenatal iron syrup supplementation had significant association with a lower probability for a low birth weight. Other variables include urban residence and antenatal attendance also had a strong influence with a low chance of low birth weight, but they were not significant in this study.

Our findings also revealed that the childhood undernutrition and disease had significant association with higher likelihood for the low birth weight. Evidence had showed that child birth defects may be genetically induced or evironmental inherent. A recent study had reported that an underweight mother had a higher risk of giving birth to an underweight baby. Our result corroborates the recent study by Kodzi et al. [25]. A meta-analysis study further gave evidence that the children born of underweight tend to have cognitive disabilities and a lower intelligence quotient [26]. Those children that surivived the episode of low birth weight had a higher risk of high blood pressure, diabetes and heart diseases at adulthood [27].

In addition, poverty and low maternal education contributes significantly to poor birth outcomes. Our report corroborated the study on the adverse effect of poor maternal socio-economic factors on low birth weight in India and Sub-Saharan Africa [28,29].

This study also revealed that firewood/dung cooking and mother smoking (tobacco) are critical risk factors of low birth weight in Nigeria. This result is complementary to early work conducted in Zimbabwe. They asserted that low birth weight not only caused by lack of socioeconomic resources but by the use of inferior energy sources for indoor cooking and air pollution [30]. Other previous studies had enunciated that early childbearing, inadequate access to prenatal health services and less disadvantage groups experienced a higher prevalence of low birth weight in cities than in rural areas in sub-Saharan Africa [31,32].

Our result gave a strong indication that iron syrup supplementation during pregnancy yields improvement in child birth weight, while postnatal vitamin A intervention would boost the child growth of those children born with low birth weight. There was a wealthy of evidence that zinc supplementation reduces diarrhea morbidity and respiratory infections among children [33,34]. Other reports from Peru and Bangladesh gave complimentary results that Zinc supplementation during antenatal resulted in an improvement in featal growth and birth weight [35,36]. In contrast, a report in China on multiple micro-nutrient during pregnancy improved birth weight, but had no effect on early neonatal mortality [37]. A contrary result was reported from Nepal that where multiple supplementation (addition of zinc to iron + folic acid) nullified the beneficial effect of iron and folic acid on birth weight in their studies [38,39]. Similarly, a study conducted in Tanzania found strong association in multiple micronutrient and reduction in the risk of perinatal mortality [40].

Our nonlinear of metrical covariates-(mother’s weight, mother’s age, mother nutritional status (body mass index)- show discernible association with the child birth weight. Our findings are consistent with established theory and even strengthened the empirical results from similar studies. Our result revealed that mother body mass index (mbmi <18.5 kg/m${}^{2}$, underweight mother) posess a higher risk of low birth weight and mbmi ≥26 kg/m${}^{2}$, obesed mother) had higher probability of giving birth to an overweight baby, and the child is at risk of being obesed. This result is consistent with a recent study by McDonald et al. [41]. Previous studies have identified that mother nutritional status (BMI) has strongly related to reproductive health outcome. Epidemiological studies had also estimated that the environmental impacts contribute about 25% birth weight variances and genetic influences accounted for between 38% to 80% birth weight variance [42,43].

In our analysis, we have established a substantial evidence of geographical variations in the birth weight of babies across states using 2008 Nigeria DHS data. This output on regional variation has corroborated the finding in similar research studies. Recent studies had showed remarkable variations in the incidence of low birth weight with geographical patterns [24,44]. It is been reported that over 20 million global infants, constituting 15.5 per cent of all births, are born with low birth weight, 95.6% of them in developing countries. The prevalence of low birth weight can be attributed to poverty level in developing countries (16.5%), which is more than double the level in developed regions (7%). Half of all low birth weight babies are born in South Central Asia, where more than a quarter (27%) of all infants weights less than 2500 grams are recorded at birth [44]. The Low birth weight levels in sub-Saharan Africa are found to be around 15% and Central and South America was about (10%). The low birth weights in the Caribbean are found to be around (14%) and almost as high as in sub-Saharan Africa [45,46]. A significant geographical variation that characterized the incidence of low birth weight in Europe, with lower rates in the more northerly countries is reported in recent study by Skokić et al. [32].

## 5. Limitations

This is cross-sectional data and is self-reported, as is the case for many survey data. Thus, the casual relationship may be difficult to establish with non-response cases. The information provided is also self-reported, so there is tendency of recall-error or bias especially with issues relating to age, birth size or actual birth weight of the children. In spite of these limitations, the authors believe that the findings from this study will facilitate targeting interventions and policy strategies.

## 6. Conclusions

In this study, we have explored a robust and flexible methodological approach to analyze the birth weights of under-five children in 2008 Nigeria Demographic and health survey data. Our multivariate analysis takes into account the influence of contextual variables on birth weight from individual household health outcomes, which can engender an important health policy making. Having controlled the confounding factors, our approach produced spatial maps of the residual effects that were not captured by the underlying factors, which would have been neglected in a classical regression settings. The statistical significance of the variables discussed in the fixed effect Tables can be used to formulate programmes for interventions and policy. The spatial plots highlight hot-spots that can assist government to channel resources in an efficient manner.

## Figures and Tables

**Table 1:** Frequency distribution of child birth weights by size category in the 2008 Nigeria Demographic and Health Survey (NDHS).

Birth Size	Birth Weight	Response	Frequency	Percent
	Intervals (in kg)			
Low	<2.5	1	4239	14.82
Average	2.5–3.2	2	10,732	37.46
Large	3.3–4.0	3	7852	27.41
Very large	>4.0	4	5160	18.11
Total			27,983	97

**Table 2:** Frequency distribution of some categorical covariates by Two-way classification and Bivariate analysis.

	Low Size	Normal Size	
	cbw<2500 (%)	cbw≥2500 (%)	*p*-Value
North Central	463 (22.5)	4534 (17.3)	<0.001 **
North East	306 (14.8)	6197 (23.7)	0
North West	367 (17.8)	7528 (28.7)	0
South East	221 (10.7)	2191 (8.3)	0
South South	269 (13.1)	2974 (11.3)	0
South West	434 (21.1)	2850 (10.8)	0
*Child’s sex*			
Female	1977 (47.0)	12,302 (52.0)	<0.001 **
Male	2262 (53.0)	11,442 (48.0)	
*Residence*			
Rural	3320 (78.0)	17,231 (73.0)	<0.001 **
Urban	919 (22.0)	6513 (27.0)	0
*Birth Types*			
Single	4005 (94.5)	23,068 (97.0)	<0.001**
Twin	234 (5.5)	676 (2.8)	00
*Birth order*			
1st order	803 (19.0)	4412 (19.0)	<0.001 **
2nd order	660 (16.0)	4195 (18.0)	
3rd order	586 (14.0)	3703 (16.0)	
4th order	523 (12.0)	3111 (13.0)	
≥5th order	1667 (39.0)	8323 (35.0)	
*Mother education*			
Imcomplete Primary	2772 (65.4)	11,275 (47.0)	<0.001 **
Primary	765 (18.0)	5646 (23.8)	
Secondary	591 (13.9)	5617 (23.7)	
High	111 (2.6)	1206 (5.1)	
*Mother employed*			
Not	1522 (36.0)	7279 (31.0)	<0.001 **
Yes	1813 (43.0)	11,773 (50.0)	
*Wealth Index*			
Poorest	1490 (35.1)	5982 (25.2)	<0.001 **
Poor	1103 (26.0)	5584 (23.5)	
Average	756 (17.8)	4684 (19.7)	
Rich	529 (12.5)	4123 (17.4)	
Richest	361 (8.5)	3371 (14.2)	
*Diarrhea*			
No	3079 (72.0)	18,967 (79.9)	<0.001 **
Yes	512(12.1)	2108 (8.9)	
*Fever*			
No	2988 (70.0)	17,745 (75.0)	<0.001 **
Yes	604 (14.0)	3317 (14.0)	
*Cough*			
No	3089 (73.0)	18,615 (78.0)	<0.001 **
Yes	502 (12.0)	2420 (10.0)	
*Vitamin A Supp.*			
No	2219 (52.0)	11,257 (47.0)	<0.001 **
Yes	433 (10.0)	3547 (15.0)	
*Antenatal*			
No	1656 (39.0)	6683 (28.0)	<0.001 **
Yes	1003 (24.0)	8224 (35.0)	
*Stunted*			
No	1448 (34.0)	10,125 (43.0)	<0.001 **
Yes	1217 (29.0)	6026 (25.0)	
*Wasted*			
No	1639 (39.0)	11,764 (50.0)	<0.001 **
Yes	1026 (24.0)	4387 (18.0)	
*Underweight*			
No	2230 (52.0)	14,142 (59.0)	<0.001 **
Yes	435 (10.0)	2011 (8.5)	
*Iron*			
No	1656 (39.0)	6683 (28.0)	<0.001 **
Yes	1003 (24.0)	8224 (35.0)	

The *p*-value marked with ** indicates that the variable was significant at 1% level. All *p*-values correspond to Pearson Chi-square test of contingency.

**Table 3:** Descriptive summary of the socio-demographic characteristics (covariates) used in the model by four-way category of birth size in 2008 NDHS.

Covariates	Four-Way Category of Child Birth Weight				
	Low	Average	Large	Very Large	Total
	N (%)	N (%)	N (%)	N (%)	N
*Zones*					
North Central (NC)	772 (18.2)	1638 (15.3)	1286 (16.4)	1220 (23.6)	5046
North East (NE)	1255 (29.6)	2670 (24.9)	1467 (18.7)	1062 (20.6)	6559
North West (NW)	1270 (30.0)	2744 (25.6)	2343 (29.8)	1372 (26.6)	7947
South East (SE)	293 (6.9)	1147 (10.7)	665 (8.5)	281 (5.4)	2450
South South (SS)	292 (6.9)	1389 (12.9)	934 (11.9)	629 (12.2)	3327
South West (SW)	357 (8.4)	1144 (10.7)	1157 (14.7)	596 (14.6)	3318
*Place of residence*					
Rural	3320 (78.0)	7825 (73.0)	5645 (72.0)	3761 (73.0)	21,034
Urban	919 (22.0)	2907 (27.0)	2207 (28.0)	1399 (27.0)	7613
*Sex of child*					
Male	1977 (46.6)	5373 (50.1)	4083 (52.0)	2846 (55.2)	14,604
Female	2262 (53.4)	5359 (49.9)	3769 (48.0)	2314 (44.8)	14,043
*Child birth type*					
Singleton	5064 (98.1)	4005 (94.5)	10,364 (96.6)	7640 (97.3)	27,685
Twin	96 (1.9)	234 (5.5)	368 (3.4)	212 (2.7)	962
*Malaria drug during pregnancy*					
No	1656 (39.0)	3115 (29.0)	2180 (28.0)	1388 (27.0)	8420
Yes	1003 (24.0)	3479 (32.0)	2775 (35.0)	1970 (38.0)	9295
*Child birth order*					
1st order	803 (19.0)	1975 (18.0)	1495 (19.0)	942 (18.0)	5353
2nd order	660 (16.0)	1901 (18.0)	1406 (18.0)	888 (17.0)	4969
3rd order	586 (14.0)	1670 (16.0)	1219 (16.0)	814 (16.0)	4388
4th order	523 (12.0)	1426 (13.0)	1005 (13.0)	680 (13.0)	3712
5th order	1667 (39.0)	3760 (35.0)	2727 (35.0)	1836 (36.0)	10,225
*Child Spacing (within 3 years)*					
<2 child	3305 (78.0)	8572 (80.0)	6413 (82.0)	4209 (82.0)	22,950
≥2	934 (22.0)	2160 (20.0)	1439 (18.0)	951 (18.0)	5697
*Wealth Index*					
Poorest	1490 (35.1)	2971 (27.7)	1913 (24.4)	1098 (21.3)	7604
poor	1103 (26.0)	2581 (24.0)	1810 (23.1)	1193 (23.1)	6871
Middle	756 (17.8)	2002 (18.7)	1502 (19.1)	1180 (22.9)	5609
Richer	529 (12.5)	1793 (16.7)	1409 (17.9)	921 (17.8)	4755
Richest	361 (8.5)	1385 (12.9)	1218 (15.5)	768 (14.9)	3808
*Under-nutrition*					
*Stunting*					
Not	1448 (34.2)	4442 (41.4)	3300 (42.0)	2383 (46.2)	11,747
Stunted	1217 (28.7)	2750 (25.6)	2010 (25.5)	1266 (24.5)	7356
*Wasting*					
Not	2831 (54.9)	1639 (38.7)	5064 (47.2)	3869 (49.3)	13,616
Yes	818 (15.9)	1026 (24.2)	2128 (19.8)	1441 (18.4)	5487
*Underweight*					
Not	2230 (52.6)	6196 (57.7)	4672 (59.3)	3274 (63.4)	16,630
underweight	435 (10.3)	997 (9.3)	639 (8.1)	375 (7.3)	2475
*Diarrhea in last 2 weeks*					
No	3079 (72.6)	8556 (74.7)	6315 (80.4)	4096 (79.4)	22,372
Yes	512 (12.1)	915 (8.5)	694 (8.8)	499 (9.7)	2645
*Fever in last 2 weeks*					
No	2988 (70.0)	7991 (74.0)	5898 (75.0)	3856 (75.1)	21,039
Yes	604 (14.0)	1482 (14.0)	1100 (14.2)	735 (14.0)	3965
*Cough in last 2 weeks*					
No	3089 (72.9)	8426 (78.5)	6182 (78.7)	4007 (77.7)	22,011
Yes	502 (11.8)	1042 (9.7)	810 (10.3)	568 (11.0)	2965
*Antenatal*					
No	1028 (24.3)	3005 (28.0)	2380 (30.3)	1776 (34.4)	8256
Yes	237 (5.6)	938 (8.7)	777 (9.9)	530 (10.3)	2500
*Vitamin A*					
No	2219 (52.0)	5175 (48.0)	3678 (47.0)	2404 (47.0)	13,591
Yes	433 (19.0)	1354 (10.0)	1234 (13.0)	959 (16.0)	4011
*Iron/Syrup Supplementation*					
No	1656 (39.0)	3115 (29.0)	2180 (28.0)	1388 (27.0)	8420
Yes	1003 (24.0)	3479 (32.0)	2775 (35.0)	1970 (38.0)	9295

**Table 4:** Descriptive statistics of the continuous covariates used in the model by Four-way category of birth size in 2008 NDHS.

Covariates	Four-Way Category of Child Birth Weight			
	Low	Average	Large	Very Large
Child weight at birth (in kg)	2.57 (0.58)	3.09 (0.59)	3.42 (0.68)	3.79 (0.79)
Mother’s age at first birth (in years)	18.7 (4.06)	19.1 (4.19)	19.4 (4.28)	19.2 (4.20)
Mother’s body mass index (mbmi)	21.7 (3.69)	22.3 (3.80)	22.6 (3.91)	22.8 (3.97)
Mother’s age, *mage* (in years)	27.1 (7.31)	27.2 (7.00)	27.5 (6.91)	27.4 (6.91)
Mother’s height (in cms)	157 (6.50)	158 (6.52)	158 (6.79)	158 (7.06)
Mother’s weight, *mwheight* (in kg)	53.9 (10.3)	55.6 (11.6)	56.5 (11.2)	57.3 (11.5)

**Table 5:** Model comparison values based on Akaike Information Criterion (AIC) and on Bayesian Information Criterion (BIC) for the three specified models together with the marginal log-likelihood (LL) and Generalized cross validation.

Model	-2LL	df	AIC	BIC	GCV
		Binary logit model			
A.1	22,449.4	35.7	22,520.7	22,814.6	0.804
A.2	4990.1	26.0	5042.1	5221.5	0.685
A.3	6052.1	55.5	6163.2	6557.6	0.676
		Multinomial logit model			
B.1	72,630.1	38.0	72,630.0	73,019.5	2.530
B.2	19,103.4	26.0	19,155.4	19,334.8	2.587
B.3	23,533.1	67.1	23,667.2	24,144.3	2.453

**Table 6:** Summary of three binary logistic models.

Variable	Model A.1		Model A.2		Model A.3	
	Est.	95% CI	Est.	95% CI	Est.	95% CI
Intercept	−1.956	(−2.156, −1.756)	−1.400	(−1.656, −1.143)	−1.193	(−1.962, −0.424)
*Geopolitical zones*						
North central (ref)	-	-	0		0	
North East (NE)	-	-	0.188	(0.023, 0.354)	0.448 *	(0.004, 0.892)
North West (NW)	-	-	−0.143	(−0.343, 0.056)	0.265	(−0.153, 0.682)
South east (SE)	-	-	0.049	( −0.162, 0.261)	−0.165	(−0.653, 0.324)
South South (SS)	-	-	−0.226	(−0.426, −0.026)	−0.390	(−0.051, 0.062)
South West (SW)	-	-	0.137	( −0.027, 0.301)	−0.228	(−0.680, 0.223)
*Child’s sex*						
Female (ref.)	-	-	0		0	
Male	-	-	0.1797 *	(0.106, 0.254)	0.171 *	(0.104, 0.237)
*Type of birth*						
Singleton (ref.)	-	-	0		0	
Multiple birth	-	-	0.206	(−0.023, 0.434)	0.377	(0.162, 0.592)
*Place of residence*						
Rural (ref.)	-	-	0		0	
Urban	-	-	−0.0114	(−0.104, 0.0810)	−0.038	(−0.124, 0.049)
*Mode of cooking*						
Cook gas, kerosene (ref.)	-	-	-	-	0	
Firewood/dung	-	-	-	-	0.068	(−0.158, 0.295)
Not literate (ref.)	-	-	0		0	
literate	-	-	−0.184	(−0.272, −0.096)	−0.118	(−0.199, −0.037)
Not (ref.)	-	-	-	-	0	
Smoke	-	-	-	-	0.474	(−0.233, 1.180)
*No sibling dead (ref.)*	-	-			0	
dead 2	-	-	-	-	0.181	(−0.034, 0.396)
dead 3	-	-	-	-	−0.369	(−0.679, −0.058)
dead 4	-	-	-	-	0.144	(−0.166, 0.454)
*Child spacing last 3 years*						
<2 birth (ref.)	-	-	0		0	
≥2 birth	-	-	0.200	(0.091, 0.308)	0.159	(0.063, 0.254)
stunted	-	-	0.047	(−0.040, 0.134)	-	-
wasted	-	-	0.226	(0.134, 0.318)	-	-
*Micro-nutrient supplementation*						
No (ref.)	-	-	0		0	
Prenatal iron/Syrup	-	-	−0.282	(−0.375, −0.189)	−0.234	(−0.318, −0.150)
*Supplementation*						
No (ref.)	-	-	0		0	
Postnatal vitamin A	-	-	0.010	(−0.073, 0.093)	−0.059	(−0.137, 0.019)
*No (ref.)*	-	-	0		0	
Antenatal	-	-	−0.083	(−0.183, 0.017)	−0.045	(−0.139, 0.050)
*Poorest (ref.)*	-	-	0		0	
Quintile 1	-	-	0.020	(−0.138, 0.178)	0.030	(−0.117, 0.178)
Quintile 2			−0.121	(−0.265, 0.023)	−0.081	(−0.216, 0.053)
Quintile 3	-	-	0.005	(−0.144, 0.154)	0.007	(−0.129, 0.143)
Quintile 4	-	-	0.051	(−0.141, 0.241)	−0.077	(−0.290, 0.137)
*No (ref.)*	-	-	0		0	
diarrhea	-	-	0.090	(−0.022, 0.203)	0.062	(−0.043, 0.167)
fever	-	-	−0.019	(−0.131, 0.093)	0.021	(−0.083, 0.125)
cough	-	-	0.059	(−0.058, 0.176)	−0.022	(−0.132, 0.089)

- = indicates the corresponding variable was not included in the model; * = significant at 5% probability level.

**Table 7:** Summary of of three Multinomial logit models.

Variable	Model B.1		Model B.2		Model B.3	
	Est.	(95% CI)	Est.	(95% CI)	Est.	(95% CI)
Threshold						
$\theta_{1}$: low birth size	−1.899	(−1.94, −1.86)	−1.089	(−1.614, − 0.564)	−1.760	(−1.97, −1.550)
$\theta_{2}$: average size	0.096	( 0.07, 0.12)	−0.513	(−1.037, 0.011)	0.350	(0.14, 0.550)
$\theta_{3}$: large size	1.551	(1.52, 1.58)	1.037	(0.513, 1.562)	1.812	(1.61, 2.020)
*NC (reference)*						
NE	-	-	−0.140 *	(−0.240, −0.039)	0.301	(−0.165, 0.770)
NW	-	-	−0.176 *	(−0.287, −0.065)	0.473 *	(0.020, 0.930)
SE	-	-	0.249 *	(0.133, 0.365)	−0.630 *	(−0.618, 0.280)
SS	-	-	0.100	(−0.004, 0.203)	0.054	(−0.422, 0.530)
*Female (reference)*						
male			0.040	(−0.002, 0.081)	−0.178 *	(−0.221, −0.130)
*Single birth (reference)*						
Multiple birth			0.078	−0.081, 0.238)	−0.271 *	(−0.434, −0.110)
*No (ref.)*		0		0		
stunted	-	-	0.025	(−0.025, 0.076)	−0.014	(−0.066, 0.040)
wasted	-	-	−0.001	(−0.057, 0.056)	−0.176 *	(−0.235, −0.121)
underweight	-		-	-	-	-
*Birth Order 1 (ref.)*			0		0	
Order 2	-	-	-	–	0.017	(−0.075, 0.110)
Order 3	-	-	-	–	0.040	(−0.053, 0.130)
Order 4	-	-	-	–	0.001	(−0.098, 0.100)
Order 5	-	-	-	–	0.032	(−0.049, 0.110)
*Rural (reference)*			0		0	
Urban			0.057 *	(0.005, 0.109)	−0.035	(−0.090, 0.02)
*Child spacing last 3 years*						
<2 birth (ref.)	-	-	0		0	
≥2 birth			−0.056	(−0.123, 0.011)	−0.103 *	(−0.173, −0.03)
*No Supplementation (ref.)*				0	0	
Iron/Syrup during pregnancy			0.090 *	(0.031, 0.148)	0.052	(−0.010, 0.11)
Postnatal Vitamin A	-	-	−0.064 *	(−0.110, −0.018)	0.096 *	(0.048, 0.14)
*No Antenatal (reference)*			0		0	
Antenatal	-	-	−0.025	(−0.079, 0.028)	0.016	(−0.041, 0.07)
*Poorest(reference)*						
Quintile 1 (poor)	-	-	−0.010	(−0.103, 0.084)	−0.026	(−0.123, 0.07)
Quintile 2 (middle)	-	-	−0.057	(−0.138, 0.024)	0.037	(−0.047, 0.12)
Quintile 3 (rich)	-	-	0.022	(−0.061, 0.106)	0.048	(−0.039, 0.13)
Quintile 4 (richest)	-	-	0.053	(−0.050, 0.156)	0.074	(−0.039, 0.19)
*No disease (ref.)*			0		0	
diarrhea	-	-	−0.052	(−0.121, 0.017)	0.029	(−0.042, 0.10)
fever	-	-	−0.004	(−0.068, 0.060)	−0.046	(−0.111, 0.02)
cough	-	-	−0.007	(−0.075, 0.060)	0.019	(−0.051, 0.088)
*Not smoking (ref.)*			0		0	
Mother smoke	-	-	−0.087	(−0.581, 0.407)	-	-
*Not literate (ref.)*						
literate	-	-	0.0112	(−0.038, 0.0609)	0.083 *	(0.030, 0.140)

- = indicates the corresponding variable was not included in the model; CI = confident interval; * significant at 5% significance level.

## Abbreviations

The following abbreviations are used in this manuscript:

MDPI	Multidisciplinary Digital Publishing Institute
NHDS	Nigeria Demographic and Health survey
U5	under five
STAR	Structured Additive regression
GAM	Generalized Additive model
AIC	Akaike Information Criteria
BIC	Bayesian Information Criteria
GVC	Generalized Cross Validation
